# Michigan State Veterinary Medical Association

**Published:** 1895-02

**Authors:** Wm. Jopling

**Affiliations:** Secretary


Secretary.

### MICHIGAN STATE VETERINARY MEDICAL ASSOCIATION.

The thirteenth annual meeting of the Michigan State Veterinary Medical Association was held in the parlors of the Hudson House, Lansing, Mich., February 5, 1895. The meeting was called to order at 1.30 P.M., the President, Dr. J. C. Whitney, of Hillsdale, in the chair.

Secretary Jopling called the roll, and the following answered to the call: Drs. J. Hawkins, Detroit; D. Cummings, Port Huron; J. A. Dell, Ann Arbor; S. Brenton, Detroit; E. A. A. Grange, Lansing; J. W. Ferguson, Bay City; B. C. McBeth, Battle Creek; J. C. Whitney, Hillsdale; Wm. Jopling, Owosso; Geo. C. Moody, Mason; W. W. Thorburn, Lansing; J. J. Jay, Detroit; A. Campbell, Jackson; James Drury, Lansing; W. M. Burdick, Chesaning; H. M. Gohn, St. Johns; J. D. Rutherford, Detroit; and A. McKercher, Lansing.

The minutes of the last meeting were read and approved.

President J. C. Whitney delivered a short address of welcome in which he thanked the Association for the honor conferred upon him in electing him President, and also made some timely suggestions for the welfare of the Association. His remarks were encouraging for the prosperity of the Association, and he called special attention to the necessity of members showing greater willingness to furnish essays and papers for the meetings in the future than had been evidenced in the past.

The following applications for membership were filed: J. D. Rutherford, M.D., V.S., Detroit; Ontario Veterinary College. Vouchers: S. Brenton and W. A. Giffen. Wallace McQueen, V.S., Oxford; Ontario Veterinary College. Vouchers: L. D. LeGear and H. M. Gohn. After proper examination they were duly elected to membership.

The Secretary's annual report showed that there were about sixty members enrolled and that the affairs of the Association were in a satisfactory condition.

The question of legislation caused considerable debate—some favoring presenting a bill to the Legislature now in session at Lansing, while others thought action had better be deferred until such time as all districts and localities in the State were better supplied with qualified veterinarians than is the case at present. The latter opinion prevailed, and action was deferred at the present time.

During the evening session Prof. E. A. A. Grange, State Veterinarian, entertained the Association in a very pleasant and profitable way on various subjects, including a talk on "Parasitic Diseases Attacking Sheep in Michigan." He presented several specimens of parasites to illustrate, in a more practical way, his discourse. He also gave some new ideas in regard to dislocation of the patella, and finally closed with a talk on "Tuberculosis," giving his experience with tuberculin as an aid in the diagnosis of tuberculosis. Dr. LeGear, who was to read a paper on "Tuberculosis" was detained at home by illness.

A general discussion followed Professor Grange's talk, and much useful information was the result.

Dr. J. A. Dell, of Ann Arbor, read the report for the Committee on Intelligence and Education.

Dr. Grange, Chairman of the Committee on Diseases, stated that the State was very free from contagious diseases at present, and had been for the past year.

Communications were received and read from Dr. W. Horace Hoskins, President United States Veterinary Medical Association, wishing the Association a successful and prosperous meeting, and asking about a resolution passed by the Michigan Association at its last meeting forbidding members of the Association to deliver lectures to any organized body of masters or journeymen horseshoers; and from J. C. Buckley, Detroit, Mich., President of the Horseshoers' National Protective Association, calling attention to their organization, stating its objects, and asking the good will of the veterinarians of the State in their behalf.

Action was taken by the Association whereby an effort will be made to have an objectionable clause removed from the Code of Ethics.

Election of officers resulted as follows: *President*, George C. Moody, Mason; *Vice-Presidents*, A. Campbell, Jackson; W. W. Thorburn, Lansing; H. M. Gohn, St. Johns; *Secretary and Treasurer*, Wm. Jopling, Owosso. *Directors:* J. D. Rutherford, Detroit; J. J. Jay, Detroit; C. W. Stowe, Saginaw; Jas. Drury, Lansing; J. W. Ferguson, Bay City; D. Cummings, Port Huron. Committee on Intelligence and Education, J. D. Rutherford, D. Cummings, H. M. Gohn. Committee on Diseases, E. A. A. Grange, J. Hawkins, J. A. Dell. Committee on Finance, A. Campbell, Jas. Drury, W. W. Thorburn.

A committee, consisting of Drs. Rutherford and S. Brenton, was appointed to draft suitable resolutions on the death of the late Dr. R. E. Reycraft, of Detroit.

Meeting adjourned at 1.30 A.M. WM. JOPLING,
Secretary.

